# Supplementary material for: A Familial Novel Putative-Pathogenic Mutation Identified in Plaque-Psoriasis by a Multigene Panel Analysis
Source: Int J Mol Sci. 2023 Mar 1;24(5):4743. doi: 10.3390/ijms24054743 (PMC10003515; doi:10.3390/ijms24054743)
Supplement: Supplementary file 1 [file ijms-24-04743-s001.zip › Supplementary Material.pdf]

A familial novel putative-pathogenically mutation identified in plaque-psoriasis by a multigene panel analysis

Marcella Nunziato[1,2†], Anna Balato[3], Anna Ruocco[1,2†], Valeria D'Argenio[1,2,4], Roberta Di Caprio[5], Nicola Balato[6], Fabio Ayala[7] and Francesco Salvatore[1,2*]

[1]CEINGE-Biotecnologie Avanzate Franco Salvatore, via Gaetano Salvatore 486, 80145 Naples, Italy
[2]Department of Molecular Medicine and Medical Biotechnologies, University of Naples Federico II, via Sergio Pansini 5, 80131 Naples, Italy
[3]Dermatology Unit, University of Campania "Luigi Vanvitelli", 80131 Naples, Italy
[4]Department of Human Sciences and Quality of Life Promotion, San Raffaele Open University, 00166 Roma, Italy.
[5]Microbiology and Virology unit, Cotugno Hospital, AORN dei Colli, Via Gaetano Quagliariello, 54, 80131, Naples, Italy
[6] Italian "School of Psoriasis" Association, 81031 Aversa, Italy
[7]Department of Clinical Medicine and Surgery, University of Naples Federico II, Naples, Italy via Sergio Pansini 5, 80131 Naples, Italy

[†]These authors contributed equally to this work.
[*]Corresponding Author

Supplementary Table S1. List of the genes included in our custom multigene panel, and their chromosomal localization.

N°	Name	Chromosome	N°	Name	Chromosome	N°	Name	Chromosome	N°	Name	Chromosome
1	*ACE*	chr17	25	*HLA-C*	chr6	49	*IVL*	chr1	73	*RNF114*	chr20
2	*ADAM33*	chr20	26	*HLA-DQA1*	chr6	50	*KIR2DS1*	chr19	74	*S100A12*	chr1
3	*ADRB2*	chr5	27	*HLA-DQB1*	chr6	51	*LCE3B*	chr1	75	*S100A7*	chr1
4	*AGER*	chr6	28	*HLA-DRB1*	chr6	52	*LCE3C*	chr1	76	*S100A8*	chr1
5	*APOE*	chr19	29	*HTR2A*	chr13	53	*MGST2*	chr4	77	*S100A9*	chr1
6	*CARD14*	chr17	30	*IFIH1*	chr2	54	*MICA*	chr6	78	*SLC12A8*	chr3
7	*CCHCR1*	chr6	31	*IFNG*	chr12	55	*MIF*	chr22	79	*SLC6A4*	chr17
8	*CCL2*	chr17	32	*IL10*	chr1	56	*MMP2*	chr16	80	*SLC9A3R1*	chr17
9	*CD226*	chr18	33	*IL12B*	chr5	57	*MTHFR*	chr1	81	*SPRR1A*	chr1
10	*CDKAL1*	chr6	34	*IL13*	chr5	58	*NAT2*	chr8	82	*SPRR1B*	chr1
11	*CDSN*	chr6	35	*IL15*	chr4	59	*NAT9*	chr17	83	*SPRR2A*	chr1
12	*CSTA*	chr3	36	*IL18*	chr11	60	*NFKB1*	chr4	84	*STAT4*	chr2
13	*CX3CR1*	chr3	37	*IL19*	chr1	61	*NFKBIA*	chr14	85	*TAP1*	chr6
14	*DEFB103A*	chr8	38	*IL1B*	chr2	62	*NOS3*	chr7	86	*TCF19*	chr20
15	*DEFB103B*	chr8	39	*IL2*	chr4	63	*PGLYRP3*	chr1	87	*TAP2*	chr6
16	*DEFB104A*	chr8	40	*IL20*	chr1	64	*PGLYRP4*	chr1	88	*TNF*	chr6
17	*DEFB104B*	chr8	41	*IL20RA*	chr6	65	*POU5F1*	chr6	89	*TNFAIP3*	chr6
18	*DEFB4A*	chr8	42	*IL23A*	chr12	66	*PRINS*	chr10	90	*TNIP1*	chr5
19	*DEFB4B*	chr8	43	*IL23R*	chr1	67	*PRR9*	chr1	91	*TRAF3IP2*	chr6
20	*ERAP1*	chr5	44	*IL28RA*	chr1	68	*PSORS1C1*	chr6	92	*TYK2*	chr19
21	*FLG*	chr1	45	*IL4*	chr5	69	*PSORS1C2*	chr6	93	*VDR*	chr12
22	*FLT4*	chr5	46	*IL4R*	chr16	70	*PSORS1C3*	chr6	94	*VEGFA*	chr6
23	*FOXP3*	chrX	47	*IL6*	chr7	71	*PTPN22*	chr1	95	*ZAP70*	chr2
24	*HLA-B*	chr6	48	*IRF2*	chr4	72	*REL*	chr2	96	*ZNF750*	chr17

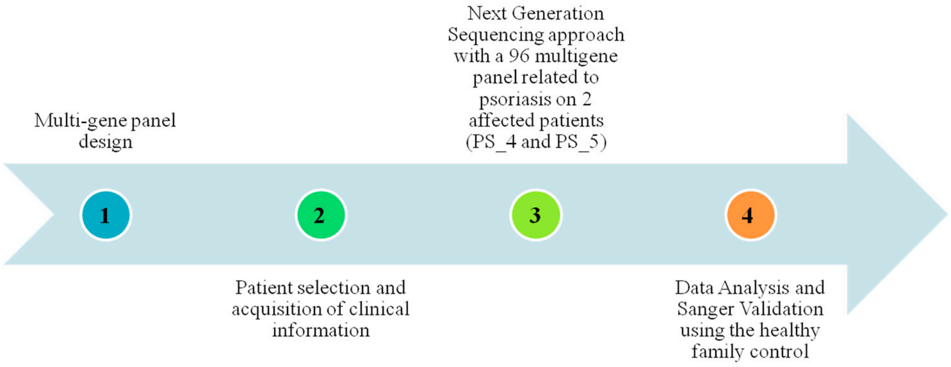

Supplementary Figure S1. The experimental protocol used in this study.
